# Machine learning photodynamics reveal intersystem-crossing-driven ladderdiene ring opening†

**DOI:** 10.1039/d4sc07395a

**Published:** 2025-06-16

**Authors:** Zhendong Li, Haijun Fu, Steven A. Lopez, Jingbai Li

**Affiliations:** a Hoffmann Institute of Advanced Materials, Shenzhen Polytechnic University 7098 Liuxian Blvd, Nanshan District Shenzhen 518055 People's Republic of China lijingbai@szpu.edu.cn; b School of Chemistry and Chemical Engineering, Zhejiang Sci-Tech University Hangzhou 310018 China; c Department of Chemistry and Chemical Biology, Northeastern University Boston MA 02115 USA s.lopez@northeastern.edu

## Abstract

Photochemical ring-opening reactions have become an essential tool for chemical syntheses under mild conditions with high atom economy. We propose a near-visible light-induced electrocyclic ring-opening reaction to afford cyclooctatetraene (COT) using carbonyl-functionalized tricyclooctadiene (1) based on our machine learning (ML) accelerated photodynamics simulations. Our CAM-B3LYP/cc-pVDZ calculations show that carbonyl group reduce the S_1_-energy of 1 to 3.65 eV (340 nm) from 6.25 eV, approaching the visible light range. The multiconfigurational CASSCF(12,11)/ANO-RCC-VDZP calculations show small S_1_ and T_1_ energy gaps near an S_1_-minimum region. Our ML-photodynamics simulations with 1000 FSSH trajectories revealed a stepwise ring-opening mechanism of 1 from the S_1_, dominated by relatively fast S_1_/T_1_ intersystem crossings in 20 ps. The trajectories predict that the quantum yield to carbonyl-functionalized COT is 89%, suggesting the light-induced ring-opening reaction of 1 is highly efficient. This work demonstrates a predictive ML-photodynamics application for photochemical reaction design.

## Abstract 2

Photochemical ring-opening reactions have become an essential tool for chemical syntheses under mild conditions with high atom economy.

## Graphical abstract

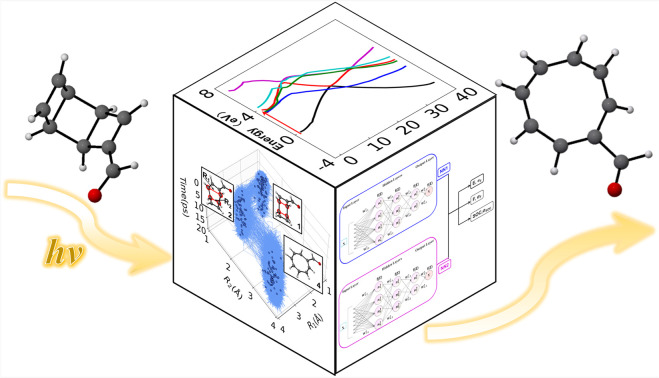

## Introduction

Cyclooctatetraene (COT) is an essential molecular framework with an eight-membered ring with four π_CC_-bonds. The unique chemical structure of COT can access versatile chemical products *via* ring-closing and cycloaddition reactions, which have been widely used in natural product synthesis,^1,2^ drug design,^3^ and organic semiconductors.^4^COT features a low-lying triplet state (1.03 eV),^5^ notable triplet absorption (350 nm),^6^ and a short triplet lifetime (100 μs).^6^ Moreover, the triplet COT exhibits relatively low reactivity towards molecular oxygen. These features make COT an excellent triplet-state quencher for developing stable organic fluorophores^7^ and laser devices.^8^

**Fig. 1:**
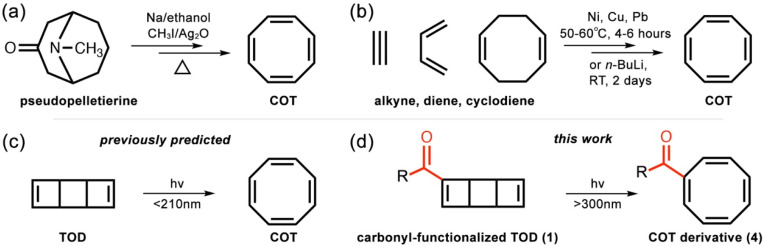
(a) Synthesis of COT*via* elimination reactions of pseudopelletierine by Willstätter. (b) Synthesis of COT with transition metal-catalyzed thermal cycloaddition of alkene, dienes, and oxidation of cyclodiene. (c) Previously predicted photochemical 4π-disrotatory electrocyclic ring-opening of TOD toward COT. (d) Proposed photochemical ring-opening of the carbonyl-functionalized TOD (1).

The first synthesis of COT was reported in 1911 by Willstätter using pseudopelletierine (Fig. 1a).^9^ Later, many efforts have been made to improve the syntheses of COT and its derivatives with ethylene,^10^ butadiene,^11,12^ and cycloocatdiene^13^ (Fig. 1b). However, most strategies require transition metal catalysts (*e.g.*, Ni,^14–16^ Cu,^11,12^ and Pb^17^) and extensive thermal energy and reaction time. Thus, a cost-efficient metal-free synthesis of COT remains a challenging task. Photochemical reactions are gaining increasing importance in organic synthesis because of the mild reaction conditions,^18^ high atomic economy,^19^ and access to exotic molecular structures.^20^ According to the literature, the photoexcitation of tricyclooctadiene (TOD) favors the electrocyclic ring-opening reactions toward COT.^21^ Our previous photodynamics studies on TOD showed the ring-opening of TOD occurs in the sub-picosecond timescale^22,23^ (Fig. 1c). Our simulations reproduced the trend of experimentally observed cubane yields depending on the substituent effects and explained the reaction pathway toward COT formation. Nevertheless, the lowest light absorption (S_1_) is far beyond the visible range (198 nm) due to the transitions to the ππ* states,^22^ which limit its applicability to produce COT.

Extending the π-conjugation of the vinyl groups with carbonyl groups to make α,β-unsaturated carbonyl compound can substantially reduce the energy S_1_ state while maintaining the photochemical reactivity.^24,25^ A recent computational and experimental study showed self-sensitized photochemical dimerization of isophorone under visible-light irradiation.^24^ These findings prompted us to envision a theoretical system featuring a carbonyl-functionalized TOD (Fig. 1d), 1 where visible light could promote the electrocyclic ring-opening reaction to generate a COT derivative (4). We are unaware of any experimental or computational studies investigating the photochemical reaction of 1. It may result from time-resolved experiments that currently cannot resolve the structural information and inform the mechanism. Carbonyl compound are known to induce intersystem crossing during the excited-state dynamics,^26,27^ which can extend the excited-state lifetime from tens to hundreds of picoseconds. The requisite multiconfigurational quantum mechanical calculations are computationally infeasible for the 10–100 s of picoseconds, even for a relatively small molecule like 1.

In this work, we combine the quantum mechanical calculations and machine learning (ML)-accelerated photodynamics approach to study the photochemical reactions of 1. We first computed the vertical excitation energy of 1 with the time-dependent density functional theory (TDDFT) and complete active space self-consistent field (CASSCF) methods. We then train neural networks (NN) based on the ground- and excited-state CASSCF energies and gradients to simulate the excited-state dynamics of 1 starting from the S_1_-Franck Condon (FC) regions. The discussion section analyzes the trajectories of possible excited-state mechanistic pathways of 1. This manuscript aims to demonstrate a promising photochemical electrocyclic ring-opening reaction of 1 for the cost-efficient and metal-free synthesis of COT under mild conditions.

## Results and discussion

### Vertical excitation

The previous study reported the lowest absorption of the unsubstituted tricyclooctadiene (TOD) at 6.25 eV (198 nm), requiring ultraviolet light for photoexcitation.^22^ Towards a more chemoselective and sustainable methodology towards COT, we introduce a carbonyl group to modulate the photophysical properties of TOD. Here, we used the aldehyde group as smallest carbonyl functional group to reduce the computational cost. Table 1 summarizes the vertical excitation energies and oscillator strength of 1 computed by various methods.

**Table 1:** The vertical excitation energies oscillator strength of 1 using different computational methods^a^

Methods	Energy (eV)						Oscillator strength		
	S_1_	S_2_	S_3_	T_1_	T_2_	T_3_	S_1_	S_2_	S_3_
CAM-B3LYP/cc-pVDZ	3.65 (nπ*)	5.14 (ππ*)	5.58 (σπ*)	2.87 (nπ*)	3.03 (ππ*)	3.74 (ππ*)	0.0006	0.0344	0.1171
CAM-B3LYP/aug-cc-pVDZ	3.68 (nπ*)	4.99 (ππ*)	5.40 (σπ*)	2.87 (nπ*)	3.09 (ππ*)	3.73 (ππ*)	0.0009	0.0384	0.1313
CAM-B3LYP/cc-pVTZ	3.70 (nπ*)	5.05 (ππ*)	5.47 (σπ*)	2.87 (nπ*)	3.10 (ππ*)	3.73 (ππ*)	0.0008	0.0355	0.1252
CASSCF(12,11)/ANO-RCC-VDZP	3.60 (nπ*)	6.92 (nπ* + ππ*)	7.45 (ππ*)	3.40 (nπ*)	3.55 (ππ*)	4.26 (ππ*)	0.00002	0.0020	0.0394
CASSCF(12,11)/ANO-RCC-VTZP	3.54 (nπ*)	6.86 (nπ* + ππ*)	7.40 (ππ*)	3.36 (nπ*)	3.52 (ππ*)	4.25 (ππ*)	0.00004	0.0017	0.0309
CASPT2 (12,11)/ANO-RCC-VDZP	3.78 (nπ*)	7.03 (ππ*^2^)	7.48 (ππ*^2^)	3.47 (nπ*)	3.74 (ππ*)	4.45 (ππ*)	0.00002	0.0021	0.0396
XMS-CASPT2 (12,11)/ANO-RCC-VDZP	4.01 (nπ*)	7.34 (ππ*^2^)	7.61 (ππ*^2^)	3.64 (nπ*)	3.92 (ππ*)	4.54 (ππ*)	0.00003	0.0065	0.0360

a The dominant electronic configurations are labelled in the parenthesis.

The CAM-B3LYP/cc-pVDZ calculations show the S_1_, S_2_, and S_3_ energies of 1 are 3.65, 5.14 and 5.58 eV, respectively. These results indicate the carbonyl group substantially lowers the S_1_ energy of 1 (3.65–3.70 eV) compared to TOD (6.25 eV).^22^ The S_1_ of 1 is a nπ* state governed by the electronic transition from the oxygen lone pair of the carbonyl group to the lowest unoccupied π*-orbital of 1 (Fig. S1†). The computed oscillator strengths (0.0006–0.0008) imply a low-intensity absorption to the S_1_ of 1 upon photoexcitation near the visible light (340 nm). The S_2_ and S_3_ are ππ* and σπ* states with larger absorption intensities and oscillator strengths than S_1_, but photoexcitation to S_2_ or S_3_ requires UV light. Including diffuse functions in the double-zeta basis set (CAM-B3LYP/aug-cc-pVDZ) shows similar excitation energies, 3.68, 4.99, and 5.40 eV, respectively. Triple-zeta basis sets (*i.e.*, CAM-B3LYP/cc-pVTZ) do not significantly affect the vertical excitation energies and characters of S_1_, S_2_, and S_3_ (3.70, 5.05, and 5.47 eV, respectively).

The triplet calculations at the CAM-B3LYP/cc-pVDZ level show nearly degenerate T_1–3_ states, where the T_1_, T_2,_ and T_3_ states are 2.87, 3.03, and 3.74 eV above S_0_, respectively. The T_1_ is a nπ* state, T2 and T3 are ππ*. The diffuse functions led to no significant effects on the triplet state energies and characters. The calculations with the cc-pVTZ basis set reproduced the similar results as cc-pVDZ. The small S_1_–T_1_ and S_1_–T_2_ gaps suggest that intersystem crossings are possible after direct photoexcitation to the FC-region.

We compared the CAM-B3LYP and ωB97X-D3 results to determine the influence of the chosen functionals on excited-state electronic structure calculations. Table S1† shows that ωB97X-D3 predicted systematically higher excited-state energies than CAM-B3LYP, which are 0.05–0.12 eV for S_1_, T_1,_ and T_2_ and 0.22–0.36 eV for S_2_ and S_3_. Despite the disagreement in excited-state energies and oscillator strengths, CAM-B3LYP and ωB97X-D3 produced the same electronic transitions in all singlet and triplet excited states (Table S2†). Thus, the CAM-B3LYP/cc-pVDZ results continue to guide our assembly of an active space for subsequent CASSCF calculations.

**Fig. 2:**
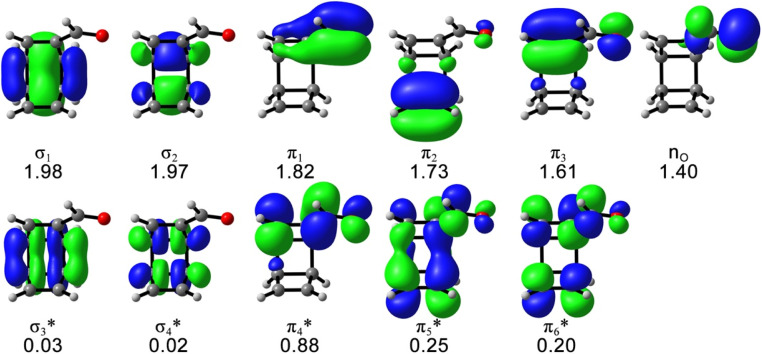
The (12,11) active space of 1 with the average occupation numbers, computed at SA5-CASSCF(12,11)/ANO-RCC-VDZP level of theory. Isosurface value = 0.04.

The possible electrocyclic ring-opening reactions of 1 after the photoexcitation to S_1_ could lead to bond-breaking, which requires multiconfigurational descriptions of the potential energy surface and possible state-crossing regions. Thus, we employed the CASSCF calculations to study the excited-state relaxation of 1 from the S_1_-FC point toward 4, including the singlet internal conversion and the singlet–triplet intersystem crossing mechanistic pathways. The active space includes 12 electrons and 11 orbitals that describe all electrons involved in the electronic excitation according to the CAM-B3LYP/cc-pVDZ calculations and the essential electrons and orbitals participating in the photochemical reactions, as shown in Fig. 2.

The S_1_ energy (3.60 eV) and electronic configuration of the 5-state-averaged (SA5)-CASSCF(12,11)/ANO-RCC-VDZP calculations agree with CAM-B3LYP/cc-pVDZ reference calculations (3.65 eV). The S_2_ and S_3_ energies are higher than the CAM-B3LYP/cc-pVDZ energies (6.92 and 7.45 eV, respectively) because the S_2_ changed to a mix of nπ* and ππ* state and the S_3_ includes double excitations in the SA5-CASSCF(12,11)/ANO-RCC-VDZP calculations. The oscillator strengths of S_1–3_ at the CASSCF(12,11)/ANO-RCC-VDZP level show the same trend as the CAM-B3LYP/cc-pVDZ results. The T_1–3_ energies from the SA5-CASSCF(12,11)/ANO-RCC-VDZP calculations are 3.40, 3.55, and 4.26 eV, respectively, which are systematically higher than the results of the CAM-B3LYP/cc-pVDZ calculations while the electronic configurations are consistent with the CAM-B3LYP/cc-pVDZ results (Table 1). We tested a larger basis set (ANO-RCC-VTZP); it does not change the results but substantially increases the computation cost by 6 times.

To verify the accuracy of the CASSCF results, we employed the state-specific and extended multistate (XMS)-CASPT2 (12,11)/ANO-RCC-VDZP calculations, which partially account for dynamical correlation. Both calculations show similar S_1_ energies to the SA5-CASSCF(12,11)/ANO-RCC-VDZP results, confirming the nπ* character. However, the S_2_ and S_3_ states show (ππ*)^2^ double excitations. Since TDDFT calculations cannot describe double excitations, it could result in discrepancies in the S_2_ and S_3_ energies compared to the CASPT2 and XMS-CASPT2 calculations. Nevertheless, the CASPT2(12,11)/ANO-RCC-VDZP and XMS-CASPT2(12,11)/ANO-RCC-VDZP calculations confirmed the relatively large S_1_–S_2_ and S_1_–S_3_ gaps (>3.0 eV) and the trends of their oscillator strengths observed in the SA5-CASSCF(12,11)/ANO-RCC-VDZP calculations. These results are in line with the CAM-B3LYP/cc-pVDZ results (1.49 eV), which avoid S_1_ state crossing with S_2_ and S_3_, especially in the FC region. The T_1–3_ states computed with the CASPT2(12,11)/ANO-RCC-VDZP and XMS-CASPT2(12,11)/ANO-RCC-VDZP calculations show similar energies and consistent electronic configurations to the SA5-CASSCF(12,11)/ANO-RCC-VDZP and CAM-B3LYP/cc-pVDZ results (Table 1). Moreover, we benchmarked the spin–orbit couplings (SOC) between S_0_, S_1_, T_1,_ and T_2_ (Table S4†). The SA5-CASSCF(12,11) calculations with the ANO-RCC-VDZP and ANO-RCC-VTZP basis sets show small differences of about 1 cm^−1^ in the SOC norms. The SA5-CASSCF(12,11)/ANO-RCC-VDZP results are consistent with the CAM-B3LYP/ZORA-TZVP calculations, including the zero-order relativistic effect. Thus, the SA5-CASSCF(12,11)/ANO-RCC-VDZP calculations are suitable for studying the photochemical reactions from the low-lying S_1_ state with the neighboring triplet states.

### Ring-opening pathways

**Fig. 3:**
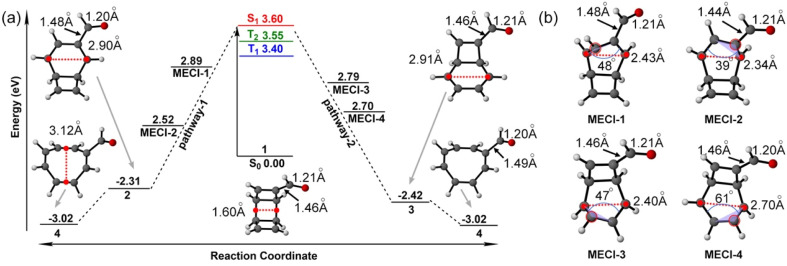
(a) Potential energy profiles for the critical structures along with two electrocyclic ring-opening pathways at the SA5-CASSCF(12,11)/ANO-RCC-VDZP level. The red dotted lines show the breaking σ_CC_ bonds. (b) Optimized structures of MECIs. The red circles highlight the pyramidalized carbon, and the blue arches show the dihedral angles of over four connected carbon atoms. The lengths of breaking σ_CC_ bonds, substitution σ_CC_ bond, and π_CO_ bonds are labelled accordingly.

Our previous work showed the electrocyclic ring-opening reaction of TOD starts from a 4π-disrotatory ring-opening process of the cyclobutene ring.^22^ We anticipate two competing reaction pathways at the carbonyl-functionalized cyclobutene ring or the unsubstituted terminus of 1. Each ring-opening pathway goes through three mechanistic critical points from the S_1_-FC point to an S_1_/S_0_ minimum energy conical intersection (MECI) towards the product. We computed the energy profiles of these critical structures in these two pathways using the SA5-CASSCF(12,11)/ANO-RCC-VDZP method to understand the ring-opening mechanism. The optimized structures and relative energies are collected in Fig. 3.

We define the reaction coordinate as the midpoint of the σ_CC_ bonds that undergo electrocyclic ring opening reactions. The electrocyclic ring-opening of 1 elongates along the reaction coordinate from 1.60 Å to 2.90 Å and 2.91 Å in two isomers (2 and 3) of the carbonyl-functionalized bicyclooctatriene (BOT) and to 3.02 Å in the product 4 (Fig. 3a). 3 is more stable than 2 because of its shorter σ_CC_ bond lengths to the carbonyl groups, which lowers the energy *via* stronger π-electron delocalization. The notable negative relative energies of 2, 3 and 4 indicate the ring-opening process of 1 is thermodynamically favored on the ground state, which provides the driving force for the ring-opening reactions.

We located two possible MECIs for each pathway. MECI-1 and MECI-2 show the breaking σ_CC_ bonds of 2.43 Å and 2.34 Å with dihedral angles of the π_CC_ bond with the carbonyl group, 48° and 39°, respectively; MECI-3 and MECI-4 show the breaking σ_CC_ bonds of 2.40 Å and 2.70 Å with dihedral angles of 47° and 61° on the other π_CC_ bond, respectively (Fig. 3b). The MECIs feature σ_CC_ bond lengths that lengthen with larger dihedral angles on the π_CC_ bonds. These dihedral angles resemble the pyramidalization of the carbon atoms in the S_1_/S_0_ conical intersection of the *cis*–*trans* isomerization of the ethylene.^28^MECI-1 show a slightly longer σ_CC_ bond with the carbonyl group (1.48 Å) than MECI-2 (1.44 Å), while the MECI-3 and MECI-4 show similar bonds of 1.46 Å due to the similar cyclobutene moieties. The π_CO_ bond distance is nearly identical (1.20–1.21 Å) in all involved structures. The MECI energies show a trend of MECI-1 (2.89 eV) > MECI-3 (2.79 eV) > MECI-4 (2.70 eV) > MECI-2 (2.52 eV). We note that the energetic trends do not correlate to the geometrical changes of the π_CC_ bond but relate to the substitution σ_CC_ bond lengths. A possible explanation is that the shortened distance to the carbonyl groups could stabilize the diradicals *via* the extended delocalization, thus lowering the MECI energy. The energetic proximity of these MECIs suggests that multiple competing mechanistic pathways may exist.

**Fig. 4:**
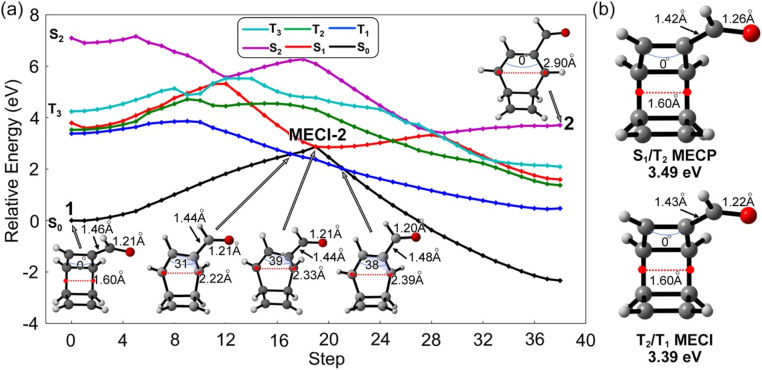
(a) Interpolated reaction pathway for the first ring-opening step of 1, computed at SA5-CASSCF(12,11)/ANO-RCC-VDZP level. The blue arches show the dihedral angles of over four connected carbon atoms. (b) Optimized structures of the S_1_/T_2_ and T_2_/T_1_ minimum energy crossing points (MECP) with the relative energies computed at the SA5-CASSCF(12,11)/ANO-RCC-VDZP level.

The S_1_-FC (3.60 eV) energy is nearly degenerate with T_2_ (3.55 eV) and T_1_ (3.40 eV) (Fig. 3a). These results prompted us to investigate S_1_/T_1_ and S_1_/T_2_ intersystem crossing mechanisms in 1. We interpolated the ring-opening pathways from the S_1_-FC to the BOT intermediates to inform the energetic relationship between the singlet and triplet states. Fig. 4a illustrates a representative potential energy curve for the three lowest singlet and triplet states from 1 to 2*via*MECI-2. The potential energy curves for other pathways show similar topology, as shown in Fig. S3.†

The interpolated structures in Fig. 4a show continuous elongations along the reaction coordinate from 1 (1.60 Å) to 2 (2.90 Å). The maximum dihedral angle of the π_CC_ bond at the MECI-2 structure is 39°. The S_1_ state shows substantially smaller gaps to T_1_ and T_2_ than S_2_ and T_3_ in the first 5 steps, suggesting the T_1_ and T_2_ states are more mechanistically relevant to the ring-opening reaction of 1 than the S_2_ and T_3_ states. An S_1_ minimum was located near the S_1_-FC point and is connected to MECI-2 through an excited-state transition structure. The increasing S_1_ energy could slow the S_1_ → S_0_ transition *via*MECI-2. On the other hand, the depth of the S_1_-minimum combined with the small S_1_/T_1_ and S_1_/T_2_ energy gaps could promote the intersystem crossing. The nearly degenerate T_1_ and T_2_ states in the S_1_ minimum region could also generate the T_2_/T_1_ internal conversion to enhance the T_1_ population. The geometry optimizations for the four interpolated pathways led to the same S_1_/T_2_ and T_2_/T_1_ minimum energy crossing points (MECPs). The S_1_/T_2_ MECP structure shows increased π_CO_ bond length (1.26 Å) and reduced length of the substitution σ_CC_ bond (1.42 Å), while the T_2_/T_1_ MECI shows a nearly identical structure to the S_0_ minimum geometry (Fig. 4b). The relative energies of the S_1_/T_2_ and T_2_/T_1_ crossings are 3.49 eV and 3.39 eV above the S_0_ minimum and under the S_1_-FC point. These results suggest the intersystem crossing the subsequent triplet internal conversions is energetically favored. The T_1_ state shows a relatively flat potential energy near the S_1_ barrier, which requires less energy than S_1_ and T_2_ to afford the ring opening of 1. The interpolated T_1_ barriers are 0.48 eV, 0.42 eV, 0.95 eV, and 0.84 eV in pathways 1–4, respectively (Fig. S3†), which are confirmed by the energy profiles at the XMS-CASPT2(12,11)/ANO-RCC-VDZP level. These results indicate pathway 2 could be the most favored reaction channel. Despite the mechanistic hints provided by this static approach, only non-adiabatic molecular dynamics can provide answers to the hypotheses stated here.

### Machine-learning photodynamic

To quantify the light-induced electrocyclic ring-opening mechanism for 1, we enumerated all possible reaction pathways from the S_1_-FC regions using nonadiabatic molecular dynamics simulations based on the SA5-CASSCF(12,11)/ANO-RCC-VDZP calculations. The simulated absorption spectrum shows notable bands in 300–400 nm because the vibronic coupling enhances the transition dipole moment of the nπ* state, suggesting the S_1_ of 1 is accessible for photoexcitation (Fig. S4†). As such, our simulations started from the S_1_-FC regions, where the initial conditions are generated by Wigner sampling at the zero-point energy level. We included S_0_, S_1_, T_1_, and T_2_ because they are involved in the ring-opening reaction of 1, according to the potential energy profile (Fig. 4). We employed the generalized fewest switches surface hopping (FSSH) method to evaluate the probability of the internal conversions and intersystem crossings.^29^ The nonadiabatic couplings (NACs) are computed by the curvature-driven time derivative coupling (*k*TDC) approach.^30,31^ The SOCs are computed with the SA5-CASSCF(12,11)/ANO-RCC-VDZP calculations. Due to the high computational cost (*e.g.*, 4 days for a single trajectory), we propagated 300 trajectories in 400 fs with a time step of 0.5 fs.

We used our open-source ML-photodynamics code Python Rapid Artificial Intelligence *Ab Initio* Molecular Dynamics (PyRAI^2^MD) to train neural networks and accelerate the CASSCF calculations of the energies, gradients, and SOCs for the considered states. The ML-photodynamic simulations allowed us to propagate about 1000 trajectories in 20 ps with a timestep of 0.5 fs at substantially reduced computational costs (*e.g.*, 16 minutes for a single trajectory instead of 200 days with SA5-CASSCF(12,11)/ANO-RCC-VDZP calculations). The NN training employed adaptive sampling to iteratively collect under-sampled data, where the unphysical structures with large energy outliers were discarded. 9% of the NN trajectories early stopped in the excited-state due to large NN prediction uncertainty, and thus are not included in subsequent discussions. The CASSCF- and NN-trajectories show similar energy conservation behavior. They maintained a small average energy drift of 0.08 and 0.01 eV, and gradually increased to 0.31 and 0.15 eV after multiple surface hoppings between discontinuous potential energy surfaces, which could be improved with reduced simulation time step.

**Fig. 5:**
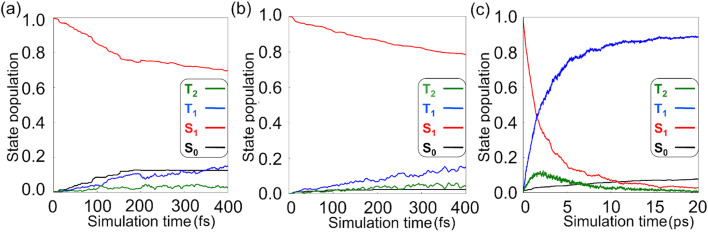
(a) The state population dynamics of 1 in 400 fs nonadiabatic molecular dynamics with the SA5-CASSCF(12,11)/ANO-RCC-VDZP calculations. (b and c) The state population dynamics of 1 in the first 400 fs and 20 ps of the ML-photodynamics simulations.

The CASSCF state populations plot in Fig. 5a shows that 69% of trajectories remained in S_1_, while the S_0_ and T_1_ populations increased to 12% and 16%, respectively. The large remaining S_1_ population suggests the reaction pathways from the S_1_-FC region are still largely unexplored. The NN-trajectories show comparable state populations to the SA5-CASSCF(12,11)/ANO-RCC-VDZP reference results (Fig. 5b). The S_1_ population reduced to 79% while the T_1_ population increased to 15%. It fits an S_1_/T_1_ intersystem crossing time constant of 1.7 ps, which is close to 2.0 ps obtained from the CASSCF reference trajectories. We note the NN-predicted trajectories of the S_1_ → S_0_ transitions are 2%, which is lower than the reference trajectories (12%).

We performed an error analysis to further determine the origin of the discrepancy between the reference and NN-predicted trajectories and possible influence on the simulations results. Fig. S5† collects the NN-predicted energies and gradients for all MECIs. The S_0_ and S_1_ of MECI-1, MECI-2, and MECI-3 show notable errors larger than 0.10 eV. As such, the enlarged out-of-sample prediction errors could lead to inaccurate population transfer from S_1_ to S_0_, resulting in an underestimated S_0_ population in the dynamics. To determine the influence of underestimated S_1_ → S_0_ transitions on the reaction channels, we turned off surface hopping (*i.e.*, infinitely slow intersystem crossing) and still observed the ring-opening reaction of 1 (Table S7†). This test suggests the ring-opening reaction of 1 is not sensitive to the underestimation of S_1_ → S_0_ transitions.

Moreover, we prepared an out-of-sample test set based on the S_1_/T_1_ surface hopping structures collected from the ML-photodynamics trajectories to inform the NN prediction accuracy for out-of-sample structures far from the equilibrium geometries. Table S8† shows that the test errors in energies, gradients, and SOC norms are 5, 2, and 6 times the validation errors. In contrast to the narrow error distributions of energies and gradients, the SOC norms show a wide error distribution from −60 to 60 cm^−1^ with an MAE of 13.4160 cm^−1^. To determine the sensitivity of ring-opening reaction to the ISC dynamics, we compared the ML-photodynamics with a constant bias by adding and subtracting the MAE to the predicted SOC norm. The 20 ps state population dynamics with reduced SOC norms (Fig. S7b†) show almost identical results to those without modifications on SOC norms (Fig. S7a†), suggesting the NN overestimations of the SOC norms do not significantly affect the S_1_ → T_1_ ISC rates. Increasing the predicted SOC norm mainly speeds up the T_1_ → S_0_ ISC after 10 ps but has little effect on the S_1_ → T_1_ ISC (Fig. S7c†). In all cases, the predicted yields of 4 are similar (Table S7†), suggesting a small influence of the NN prediction errors on the ring-opening reaction mechanism of 1. Thus, the NN errors will not significantly change our results and discussions on the photochemical ring-opening reaction mechanism of 1.

**Fig. 6:**
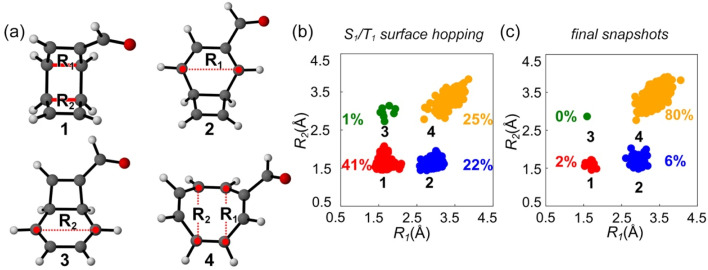
(a) Illustration of the σ_CC_ bonds *R*_1_ and *R*_2_ in 1–4. The scatter plots for the structural distributions of (b) the latest S_1_/T_1_ surface hopping points and (c) the final snapshots *via* the S_1_ → T_1_ transition in the ML-photodynamics simulations. The 2D space is defined by *R*_1_ and *R*_2_.

The NN-predicted state population dynamics in 20 ps show that 89% of the trajectories finished at the T_1_ state; only 8% trajectories arrived to S_0_ state *via* the S_1_ → S_0_ (3%), T_1_ → S_0_ (2%), T_2_ → S_0_ (3%) transitions (Fig. 5c). These results confirmed the S_1_/T_1_ intersystem crossing controls the S_1_ non-radiative relaxation of 1. We measured the σ_CC_ bond lengths in 1–4 to understand the structural changes during the S_1_ → T_1_ transitions in the ML-photodynamics simulations (Fig. 6a).

The structural distributions in Fig. 6b show four clustering regions at the latest S_1_/T_1_ surface hopping points. 41% of trajectories at region 1 correspond to the trajectories hop to T_1_ without breaking the σ_CC_ bonds. 22% of the trajectories undergo a 4π-disrotatory ring-opening at the carbonyl-functionalized cyclobutene ring at region 2; 1% of the trajectories open the unsubstituted cyclobutene ring at region 3. It suggests the carbonyl group facilitates the ring-opening reaction. Moreover, 25% of the trajectories at region 4 indicate the ring-opening reaction of two σ_CC_ bonds could also occur during the S_1_ → T_1_ transitions. These results suggest that the ring-opening reactions are intrinsically favored in the S_1_ and T_1_ states of 1.

Fig. 6c shows the final structural distributions of the trajectories undergoing the S_1_/T_1_ surface hoppings. The number of trajectories at regions 1–3 substantially decreased to 2%, 6%, and 0%, respectively. As a result, 80% of the trajectories arrived in region 4. The trajectories forming 4 remained in T_1_ after 20 ps due to slow T_1_ → S_0_ ISC. We considered them as final ground-state products since we did not observe any reverse reaction. The continuously increasing yield of 4 in our simulations suggests the second step ring-opening reaction of 1 is also thermodynamically favored.

The rare S_1_ → S_0_ conical intersection and T_*n*_ → S_0_ intersystem crossings generally begin without breaking the σ_CC_ bonds of 1. The trajectories undergoing the S_1_ → S_0_ transitions directly afforded the ring-opening reaction. The trajectories in the T_1,2_ → S_0_ pathways first showed the above-mentioned S_1_ → T_1_ or S_1_ → T_2_ transitions and continued to hop to the ground state *via* the T_1_/S_0_ or T_2_/S_0_ intersections. These trajectories collectively contribute to another 7% of 4 on the ground state. In addition, we found 2% of 4 formed in the remaining S_1_ trajectories. These results suggest even the minor reaction pathways favor the ring-opening reactions of 1. Our ML photodynamics simulations predicted an overall 89% quantum yield of 4.

**Fig. 7:**
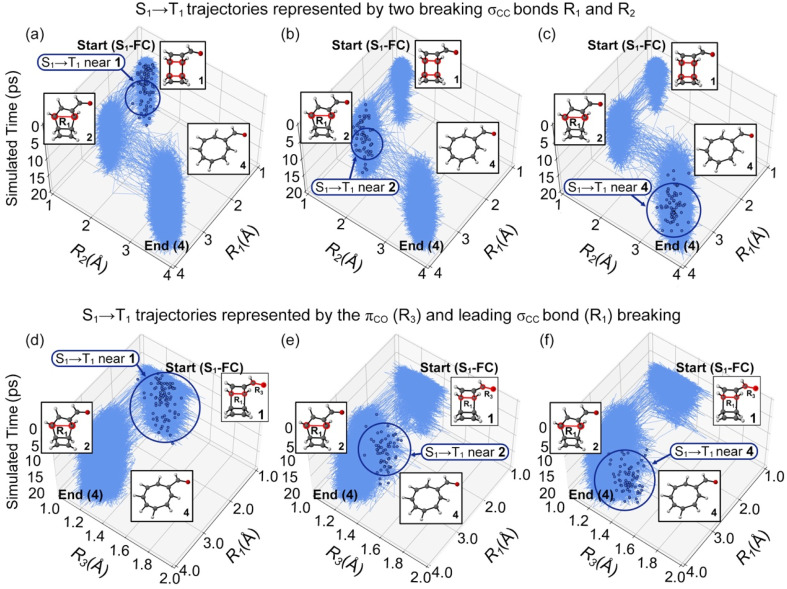
Plots for the randomly selected trajectories in the 20 ps ML-photodynamics simulations. Three top panels plot the trajectories undergoing the S_1_/T_1_ intersystem crossings at regions (a) 1, (b) 2, and (c) 4 in a space defined by intramolecular distance *R*_1_ and *R*_2_. Three bottom panels plot the trajectories undergoing the S_1_/T_1_ intersystem crossings at regions (d) 1, (e) 2, and (f) 4 in a space defined by the intramolecular distance *R*_1_ and the π_CO_ bond distance *R*_3_. The blue dots represent the latest S_1_/T_1_ surface hopping points in trajectories. The dark blue circles highlight the locations of the S_1_/T_1_ surface hopping regions.

We compared the NN-predicted trajectories in all possible reaction pathways to elucidate the ring-opening mechanisms of 1. The trajectory plots in Fig. 7a–c illustrate the changes in the σ_CC_ bond lengths, *R*_1_ and *R*_2_, as a function of simulation time.

Fig. 7a represents the trajectories undergoing the S_1_/T_1_ intersystem crossings near the S_1_-FC region at *R*_1_ = 1.62 Å and *R*_2_ = 1.64 Å (region 1). After hopping to T_1_, these trajectories proceeded to the intermediate 2 at *R*_1_ = 2.92 Å and *R*_2_ = 1.64 Å and then the final product 4 at *R*_1_ = 3.34 Å, *R*_2_ = 3.31 Å, showing a stepwise ring-opening mechanism. Fig. 7b shows the first ring-opening step near the intermediate 2 before the S_1_ → T_1_ transition. Then, the trajectories continued the second ring-opening step to form 4. Fig. 7c highlights the trajectories formed 4 before the S_1_/T_1_ intersystem crossings. These results suggest a stepwise mechanism of the photochemical ring-opening reactions of 1 that first breaks the σ_CC_ bond in the ring substituted with the carbonyl groups and then breaks the other σ_CC_ bond. The S_1_/T_1_ intersystem crossings can occur at the S_1_-FC region, intermediate 2, and product 4 indicates the σ_CC_ bond-breaking does not correlate with the S_1_/T_1_ intersystem crossing structures. We plot the π_CO_ bond distance (*R*_3_) in the carbonyl group with *R*_1_ in the trajectories (Fig. 7d–f) to determine the origin of the S_1_/T_1_ intersystem crossings. The average value of *R*_3_ is 1.22 Å at the S_1_-FC region. It notably increases to 1.31 Å, 1.30 Å, and 1.46 Å at the S_1_/T_1_ intersystem crossing structures near the S_1_-FC (Fig. 7d), intermediate 2 (Fig. 7e), and product 4 regions (Fig. 7f), consistent with a partial triplet electronic structure. Thus, the elongation of the π_CO_ bond is responsible for the S_1_/T_1_ intersystem crossings during the photochemical ring-opening reaction of 1.

**Fig. 8:**
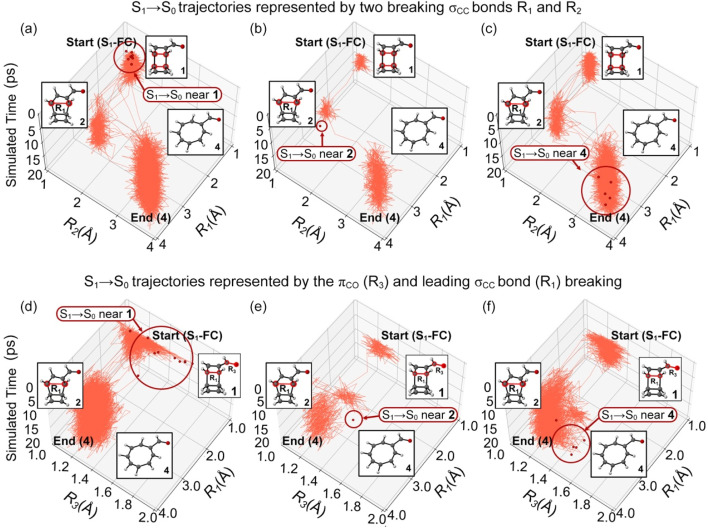
Plots for the randomly selected trajectories in the 20 ps ML-photodynamics simulations. Three top panels plot the trajectories undergoing the S_1_/S_0_ surface hoppings at regions (a) 1, (b) 2, and (c) 4 in a space defined by the intramolecular distance *R*_1_ and *R*_2_. Three bottom panels plot the trajectories undergoing the S_1_/S_0_ surface hoppings at regions (d) 1, (e) 2, and (f) 4 in a space defined by intramolecular distance *R*_1_ and the π_CO_ bond distance *R*_3_. The red dots represent the latest S_1_/S_0_ surface hopping points in trajectories. The dark red circles highlight the locations of the S_1_/S_0_ surface hopping regions.

The trajectories in Fig. 8a show the S_1_/S_0_ surface hopping structures near the S_1_-FC region (*R*_1_ = 1.66 Å and *R*_2_ = 1.66 Å). Like the trajectories hoped to T_1_ in Region 1 (Fig. 7a), these trajectories proceeded to a stepwise ring-opening reaction of 1 to form 4. Fig. 8b illustrates the S_1_ trajectories hoped to the ground state between the first and second steps of the ring-opening reaction of 1. The S_1_/S_0_ surface hopping structures resemble MECI-2 with *R*_1_ = 3.14 Å, *R*_2_ = 1.66 Å. The second ring-opening step was completed in the ground state at product 4, with *R*_1_ = 3.14 Å and *R*_2_ = 3.30 Å at the end of the simulations. The trajectories directly formed 4 in S_1_ are shown in Fig. 8c, and the trajectories still follow a stepwise ring-opening pathway. Similar to the S_1_/T_1_ intersystem crossings, we found increased π_CO_ bond lengths from 1.22 Å at the S_1_-FC regions to 1.58 Å, 1.54 Å, and 1.58 Å at the S_1_/S_0_ conical intersections near the S_1_-FC (Fig. 8d), intermediate 2 (Fig. 8e), and product 4 regions (Fig. 8f). These findings indicate the same ring-opening mechanism of 1*via* the S_1_/S_0_ conical intersections and S_1_/T_1_ intersystem crossing but only differ in the time constants.

**Fig. 9:**
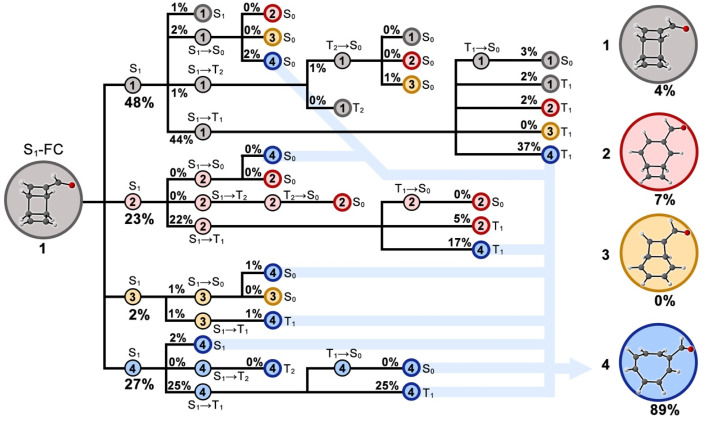
Reaction network diagram for the photochemical electrocyclic ring-opening reaction of 1. The nodes with thin black circles correspond to the intermediate species, and the terminal nodes with bold-colored circles are products 1 (gray), 2 (pink), 3 (brown), and 4 (blue). The yields less than 1% are labelled with 0%. The blue arrows indicate the sources of product 4.

Combining the electronic state population distributions and structural classifications in the trajectories produces a comprehensive reaction network for the predicted photochemical electrocyclic ring-opening reaction of 1 toward 4 (Fig. 9). The detailed and quantitative connections over all reactant, intermediates, and products across multiple singlet and triplet states provide a deep understanding of the ring-opening mechanisms.

## Conclusion

We combined the quantum mechanical calculations and machine learning photodynamics approach to enable long timescale photodynamics simulations for uncovering unexpected photochemical reaction pathways. Our showcase study on the stepwise ring-opening mechanisms of 1 show competing S_1_/T_1_ intersystem crossings and S_1_/S_0_ conical intersections, where the first ring-opening step starts at the carbonyl-functionalized cyclobutene ring. The CAM-B3LYP/cc-pVDZ calculations show that connecting to a carbonyl group substantially reduced the S_1_ energy from 6.25 eV to 3.68 eV. The SA5-CASSCF(12,11)/ANO-RCC-VDZP calculations show a close S_1_/T_1_ gap (0.2 eV) in the S_1_-FC region along with the interpolated ring-opening reaction pathways. The 20 ps ML-photodynamics simulations show dominant S_1_/T_1_ intersystem crossings with a lifetime of 1.4 ps. The overall predicted quantum yield of 4 is 89%.

The trajectory analysis revealed a diverse reaction channel of the photochemical ring-opening reaction of 1 in S_1_ and T_1_ states. The ring-opening mechanism favors a stepwise σ_CC_ bond-breaking in 1, where the cyclobutene substituted with the carbonyl group opens before the ring on the other side. Further characterizations of the trajectories show that the elongation of the π_CO_ bond length of the carbonyl groups generates the S_1_/T_1_ intersystem crossing and S1/S0 conical intersections. Thus, the photochemical ring-opening reaction of 1 is independent of whether it is in the S_1_ or T_1_ states. As such, the S_1_ excitation energy became the main driving force to break the σ_CC_ bonds of 1, which explains the high efficiency of the ring-opening reaction. Overall, our work demonstrates a feasible and light-controllable reaction tool for preparing the COT derivatives in a high yield.

## Computational details

### Quantum mechanical calculation

We optimized the gas-phase geometries of 1 using the density functional theory (DFT) at the PBE0/cc-pVDZ level. Frequency calculations confirmed the local minimum with no imaginary frequencies. The vertical excitation energies and oscillator strengths were computed at the CAM-B3LYP/cc-pVDZ, CAM-B3LYP/aug-cc-pVDZ and CAM-B3LYP/cc-pVTZ levels using the time-dependent DFT (TD-DFT) theory. The (TD)DFT calculations were carried out by the BDF program.^32–34^ The SA5-CASSCF(12,11)/ANO-RCC-VDZP, CASPT2(12,11)/ANO-RCC-VDZP, and XMS-CASPT2(12,11)/ANO-RCC-VDZP calculations were performed with OpenMolcas.19.11.^35^ The CAPST2 and XMS-CASPT2 used a level shift of 0.4 eV to avoid the intruder state problem.

### Training data generation

The initial training data consists of two parts, generated by Wigner sampling and geometry interpolations. We first generate 20 non-equilibrium structures for 1 with the Wigner sampling at the zero-point energy level. Then, we collected 39 structures in the interpolated pathway from 1 to each MECI (Fig. S3†). Mixing the Wigner sampled and interpolated structures in all four pathways gave 3210 data points. The Wigner sampling uses the PyRAI^2^MD sampling tool;^36^ the geometry interpolation uses the geodesic interpolation program.^37,38^ The training data contain the S_0_, S_1_, T_1,_ and T_2_ energies, gradients, and the norm of the spin–orbit couplings (SOCs) for S_1_–T_2_, S_1_–T_1_, S_0_–T_2_, and S_0_–T_1_, computed with the SA5-CASSCF(12,11)/ANO-RCC-VDZP calculations. We performed adaptive sampling to collect the under-sampled data points in the initial training data. Two independently trained NNs were used as a committee model. We used the potential model (*i.e.*, energies and gradients) together with the SOC model to propagate 100 trajectories from the S_1_-FC points in 10 ps with a step size of 0.5 fs. We used the standard deviations of NN-predicted energies, gradients, and SOC norms to measure the prediction uncertainty, which early stopped the trajectories when the prediction uncertainty exceeded the empirical thresholds (energy: 0.05 hartree; gradient: 0.25 hartree bohr^−1^; SOC norm: 60 cm^−1^). The final data size is 6459. Details about the adaptive sampling are available in Table S5.† All training data are computed with the SA5-CASSCF(12,11)/ANO-RCC-VDZP calculation.

### Neural networks

We implemented a fully connected feedforward NNs using TensorFlow/Keras API for Python. The NN computes the inverse distance matrix of the input molecule to predict the energies, gradients, and SOC norms. The predicted energy gap between two singlet or triplet states is used to compute the curvature-approximated time derivative coupling (*k*TDC),^30,31^ derived from the Baeck–An approximation.^39^ The NNs employ a leaky softplus activation function. The NNs used the first-order energy derivatives of atomic coordinates to fit the gradients, which ensures the physical relationship between energy and gradient. The energies and gradients of four electronic states are trained together with a combined loss function. Four NNs were trained simultaneously, where two NNs predict energy and gradient of four electronic states and two NNs predict SOC norms of four pairs of states. The training data are split into training and validation sets in a 9 : 1 ratio. The final validation mean absolute errors (MAEs) for NN-predicted energies are 0.0337–0.0340 eV. Additional information about the NN training and error evaluation is available in ESI.†

### ML-photodynamics simulations

We prepared the initial conditions for 1 by Wigner sampling at the zero-point energy level. The ML-photodynamics simulations were set to 20 ps with a timestep of 0.5 fs. The probability of a nonadiabatic electronic transition was computed with Tully's fewest switches surface hopping (FSSH) method^40,41^ using spin-diabatic representation, where the generalized formalism^29^ was used to take the intersystem crossing into account. We used the curvature-approximated time-derivative coupling (*k*TDC) method^30,31^ to evaluate the nonadiabatic couplings based on the NN-predicted energies when the S_1_–S_0_ and T_2_–T_1_ gaps are smaller than 0.5 eV. The ML-photodynamics approach is implemented in PyRAI^2^MD.^36^ The nuclear timestep is 0.5 fs and the electronic timestep is 0.025 fs corresponding to 20 substeps in the surface hopping calculations.

## Author contributions

Z. L. performed all calculations and prepared the first draft of the manuscript. Z. L. and J. L. analyzed the data. H. F. helped revise the manuscript. S. A. L. and J. L. conceived the idea of this work and revised the manuscript. J. L. oversaw the administration of this project.

## Conflicts of interest

The authors delcare no conflicts of interests.

## Supplementary Material

SC-016-D4SC07395A-s001

## Data Availability

The code for PyRAI2MD can be found at https://github.com/mlcclab/PyRAI2MD-hiam. The data supporting this article have been included as part of the ESI† and can be found at https://github.com/mlcclab/PyRAI2MD_publications/tree/main/triplet_COT.
