# Supplementary material for: Machine learning photodynamics reveal intersystem-crossing-driven ladderdiene ring opening
Source: Chem Sci. 2025 Jun 16;16(28):13031–41. doi: 10.1039/d4sc07395a (PMC12177735; doi:10.1039/d4sc07395a)
Supplement: SC-016-D4SC07395A-s001 [file SC-016-D4SC07395A-s001.pdf]

Supplementary Information (SI) for Chemical Science.
This journal is © The Royal Society of Chemistry 2025

Machine learning photodynamics reveal intersystem-crossing-driven ladderdiene ring opening

Zhendong Li,[a] Haijun Fu,[a,b] Jingbai Li,[a]* and Steven A. Lopez[b]*

[a]Hoffmann Institute of Advanced Materials, Shenzhen Polytechnic University, 7098 Liuxian Blvd, Nanshan District, Shenzhen, 518055, People's Republic of China
[b]School of Chemistry and Chemical Engineering, Zhejiang Sci-Tech University, Hangzhou 310018, China
[c]Department of Chemistry and Chemical Biology, Northeastern University, Boston, MA 02115, U.S.A.

*Corresponding to lijingbai@szpu.edu.cn and s.lopez@northeastern.edu

Table of Content

S1. Quantum mechanical calculations S2
S2. Neural networks S4
S3. Additional dynamics results S9
S4. Electronic energies and Cartesian coordinates of the optimized geometries S11
S5. References S15

S1. Quantum mechanical calculations

We compared CAM-B3LYP and ωB97X-D3 functional in the excited state calculations of **1**, which show consistent results. The ωB97X-D3 results are listed in Table S1 and the electornic configurations are shown in Table S2.

Table S1. The vertical excitation energies, oscillator strength of 1 at ωB97X-D3 level.

Method	Energy(eV)						Oscillator strength		
	S_1	S_2	S_3	T_1	T_2	T_3	S_1	S_2	S_3
ωB97X-D3/ cc-pVDZ	3.73	5.50	5.80	2.95	3.14	3.86	0.0007	0.0886	0.1141
ωB97X-D3/ aug-cc-pVDZ	3.76	5.35	5.63	2.94	3.21	3.85	0.0009	0.1064	0.1110
ωB97X-D3/ cc-pVTZ	3.77	5.40	5.69	2.92	3.21	3.82	0.0008	0.0996	0.1102

Table S2. The electronic configurations of **1** at different levels.

Method	S_1	S_2	S_3	T_1	T_2	T_3
CAM-B3LYP/ cc-pVDZ	$n_O\pi_4^*$	$\pi_3\pi_4^*$	$\sigma_2\pi_4^*$	$n_O\pi_4^*$	$\pi_2\pi_4^*$	$\pi_3\pi_5^*$
CAM-B3LYP/ aug-cc-pVDZ	$n_O\pi_4^*$	$\pi_3\pi_4^*$	$\sigma_2\pi_4^*$	$n_O\pi_4^*$	$\pi_2\pi_4^*$	$\pi_3\pi_5^*$
CAM-B3LYP/ cc-pVTZ	$n_O\pi_4^*$	$\pi_3\pi_4^*$	$\sigma_2\pi_4^*$	$n_O\pi_4^*$	$\pi_2\pi_4^*$	$\pi_3\pi_5^*$
ωB97X-D3/ cc-pVDZ	$n_O\pi_4^*$	$\pi_3\pi_4^*$	$\sigma_2\pi_4^*$	$n_O\pi_4^*$	$\pi_2\pi_4^*$	$\pi_3\pi_5^*$
ωB97X-D3/ aug-cc-pVDZ	$n_O\pi_4^*$	$\pi_3\pi_4^*$	$\sigma_2\pi_4^*$	$n_O\pi_4^*$	$\pi_2\pi_4^*$	$\pi_3\pi_5^*$
ωB97X-D3/ cc-pVTZ	$n_O\pi_4^*$	$\pi_3\pi_4^*$	$\sigma_2\pi_4^*$	$n_O\pi_4^*$	$\pi_2\pi_4^*$	$\pi_3\pi_5^*$

Table S3. The vertical excitation energies and oscillator strengths of acetyl-substituted **TOD** using CAM-B3LYP/cc-pVDZ method.

Energy(eV)						Oscillator strength		
S_1	S_2	S_3	T_1	T_2	T_3	S_1	S_2	S_3
3.83 ($n\pi^*$)	5.27 ($\pi\pi^*$)	5.74 ($\sigma\pi^*$)	3.25 ($n\pi^*$)	3.42 ($n\pi^*$)	4.16 ($n\pi^*$)	0.0007	0.0239	0.0490

Table S4. The SOC norms of acetyl-substituted **TOD** at different levels.

Method	S_0/T_1 (cm^{-1})	S_0/T_2 (cm^{-1})	S_1/T_1 (cm^{-1})	S_1/T_2 (cm^{-1})
SA5-CASSCF(12,11)/ANO-RCC-VDZP	67.0529	14.4970	8.6242	39.9552
SA5-CASSCF(12,11)/ANO-RCC-VTZP	66.0108	12.3438	7.3767	39.6072
CAM-B3LYP/cc-pVTZ	51.8468	11.7019	3.6597	18.5206
CAM-B3LYP(ZORA)/ZORA-TZVP[a]	52.4013	12.7196	4.0389	18.7300

[a]The CAM-B3LYP(ZORA)/ZORA-TZVP calculations include the zero-order relativistic effect. Overall, the SOC norms computed at the SA5-CASSCF(12,11)/ANO-RCC-VDZP level are consistent with all methods used in the benchmark. The possible overestimation of SOC norms could lead to faster singlet-to-triplet ISC than the ground truth. Nevertheless,

our test ML-photodynamics simulations suggest that the ring-opening reaction is not sensitive to minor errors in the SOC norms.

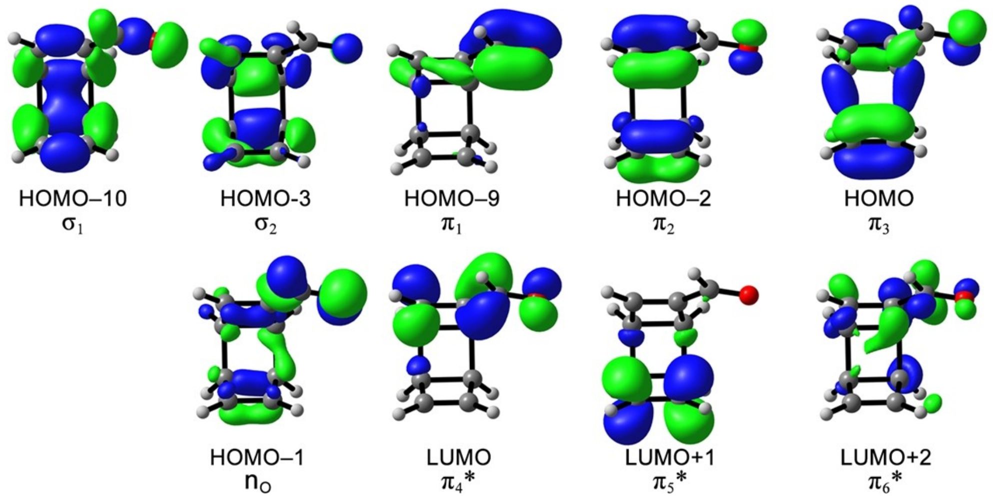

Figure S1. Molecular orbitals that participate the excitation and electrocyclic ring-opening reaction of **1** computed at CAM-B3LYP/cc-pVDZ level. Isosurface value = 0.06.

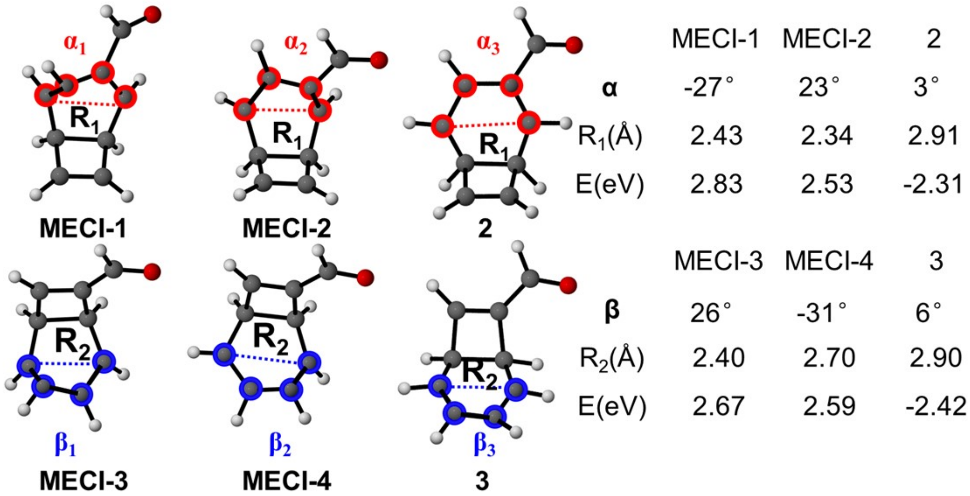

	MECI-1	MECI-2	2
α	-27°	23°	3°
R_1(Å)	2.43	2.34	2.91
E(eV)	2.83	2.53	-2.31

	MECI-3	MECI-4	3
β	26°	-31°	6°
R_2(Å)	2.40	2.70	2.90
E(eV)	2.67	2.59	-2.42

Figure S2. The geometrical parameters of MECI and intermediates, optimized with the SA5-CASSCF(12,11)/ANO-RCC-VDZP calculations.

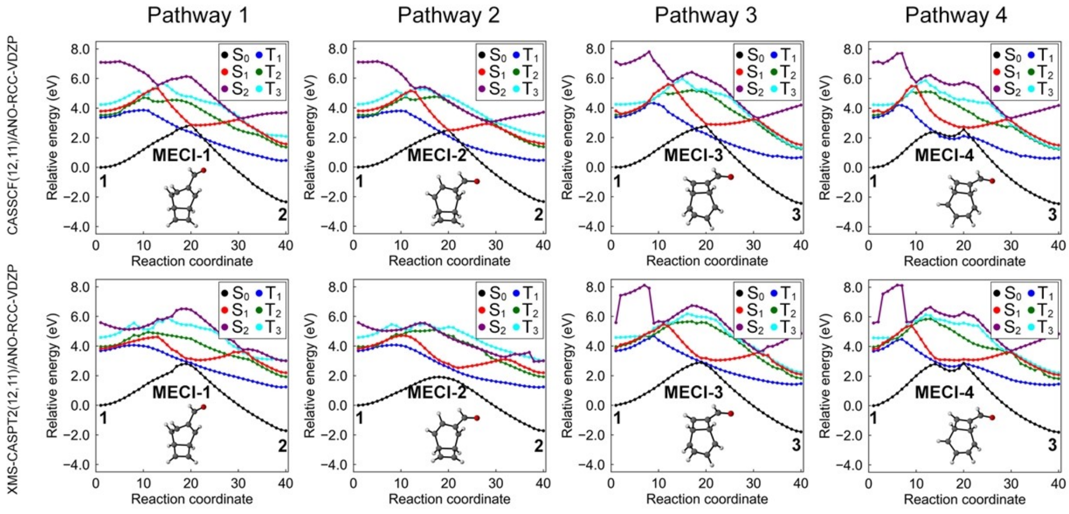

Figure S3. Interpolated excited-state pathways from **1** to **2** and **3** via multiple MECIs, computed at the SA5-CASSCF(12,11)/ANO-RCC-VDZP and XMS-CASPT2(12,11)/ANO-RCC-VDZP level.

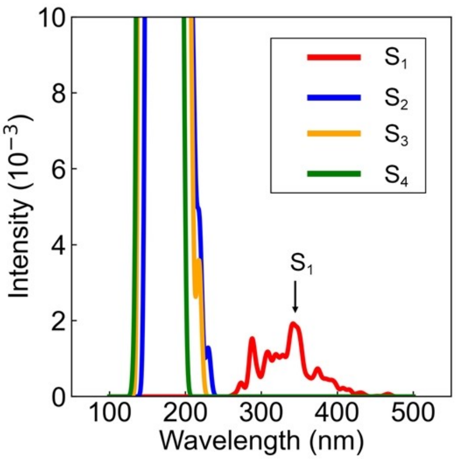

Figure S4. Simulated absorption spectrum of **1**, computed at the SA5-CASSCF(12,11)/ANO-RCC-VDZP level.

S2. Neural networks

Architecture

We implemented a fully connected feedforward NN using TensorFlow/Keras API for Python[1]. The NN computes the inverse distance matrix of the input molecule to predict the energies, gradients and the norm of the spin-orbit couplings (SOCs) of the lowest two singlet and triplet states. The predicted energy gap between two electronic states is used to compute the curvature-approximated time derivative coupling (*k*TDC)[2, 3]. The NN employs a leaky softplus activation function. The energies and forces of S_1, S_0, T_2, and T_1 are trained with a combined loss function to ensure their physical relationship. The loss function is defined as Loss = $w_E \cdot \sum(E_{pred} - E_0)^2/N + w_F \cdot \sum(F_{pred} - F_0)^2/(3N)$, where N is the number of atoms. The SOC data includes the SOC between the singlet and triplet state, S_1-T_2, S_1-T_1, S_0-T_2, and S_0-T_1. We use a learning rate scheduler that reduces the learning rate from 10^{-3} to 10^{-4} and 10^{-5} when the validation loss reaches a plateau. The training data was split into training and validation sets in a 9:1 ratio.

We optimized the NN hyperparameters based on a grid search over the number of hidden layers (3, 4, 5, 6, 7, 8, 9), the number of nodes per hidden layer (300, 400, 500, 600, 700, 800, 900), the batch size (32, 64) and the L2 regularization factor (10^{-7}, 10^{-8}, and 10^{-9}). The datasets are split in 9:1 for training, validation. The optimal values are listed in Table S5.

Table S5. Selected hyperparameters of the NN models.

Parameters				
Initial data size		3120		
Activation function		Leaky softplus		
Train: valid		9:1		
Model	EnergyGrad1	EnergyGrad2	SOC1	SOC2
Layers	8	6	7	6
Nodes	700	800	700	700
Batch size	64	64	64	64
L2 regularization	10^{-7}	10^{-9}	10^{-9}	10^{-8}
Step of learning rate		100/300/100		
learning rate		$10^{-3}/10^{-4}/10^{-5}$		

Adaptive sampling

We prepared the initial data set base on the reaction pathways shown in Figure 4 and Figure S3. In each reaction pathway, we interpolated 39 intermediate structures, mixed with 20 Wigner sampled geometries, which gave 780 structures (3120 in total). We performed the adaptive sampling to collect the undersampled structures in the initial set. Two independently trained NNs were used as a committee model. We used the potential model (i.e., energies and gradients) together with the SOC model to propagate 100 trajectories from the S_1-FC points in 10 ps with a step size of 0.5 fs. We used the standard deviations of NN-predicted energies, gradients, and SOC norms to measure the prediction uncertainty, which early stopped the trajectories when the prediction uncertainty exceeded the empirical thresholds (energy: 0.05 Hartree; gradient: 0.25 Hartree/Bohr; SOC norm: 60 cm^{-1}). The energies, gradients and SOCs of the last snapshots were recomputed with SA5-CASSCF(12,11)/ANO-RCC-VDZP calculations and added to the training data. Then we retrained the NNs to rerestart the simulations until the prediction the number of uncertain structures is satisfied. The final dataset has 6459 structures.

Table S6. Final validation MAE of the NN-predicted energies (eV), gradients (eV/Å), and SOC norm (cm^{-1}).

Model	Energy 1	Energy 2	Grad 1	Grad 2	SOC 1	SOC 2
MAE	0.0337	0.0340	0.1264	0.1356	1.9262	2.4434
R^2	0.9973	0.9979	0.9896	0.9840	0.9310	0.9128

Training four NNs (two predict the energies and gradients of four electronic states, and two predict the SOC norms of four pairs of states) simultaneously by running multiple threads on a 36-core CPU, which takes 52 minutes for 500 epochs with 91 batches. Besides training NNs, the CASSCF calculations are the main computational cost. Computing the requisite data of energies, gradients, and SOC norms at SA5-CASSCF(12,11)/ANO-RCC-VDZP took 40 min. The initial training set has 3120 data points. Since the structures are known, all data can be computed by simultaneously distributing the calculations to our computing cluster, so the wall clock time is less than 1 hour. The adaptive sampling repeatedly performed ML-photodynamics and CASSCF calculations for the collected structures. Each iteration propagated 100 single-threaded ML- photodynamics simulations on 20 CPU threads (i.e., 5 rounds) and then distributed about 100 single-threaded CASSCF calculations to 50 CPU threads (i.e., 2 rounds). A single ML-photodynamics trajectory is completed in 16 minutes, and then 5 rounds of ML-photodynamics require 80 minutes. Two rounds of CASSCF calculations need about 80 minutes. As a result, one iteration of adaptive sampling takes 3 hours, including the I/O process. The final set has 6459 data points, corresponding to 34 iterations. In rare cases, the unconverged CASSCF calculations interrupted the adaptive sampling. Therefore, adding the time to resume the adaptive sampling, the total computational cost sampling is about 5 days

The training simulations during the adaptive sampling often suffer energy conservation problems (e.g., total energy drifting of 1.35 eV after 10 ps) because of the prediction errors in energies and gradients. This is one of the purposes of finding undersample data, where the NN prediction errors become large and break energy conservation. After adaptive sampling, the 10 ps ML-photodynamics simulations show a mean absolute energy drifting of 0.15 eV after 10 ps. The remaining value of energy drifting results from the numerical errors accumulated in the kinetic energy rescaling during the frequent intersystem crossings. In the above S_1 and T_1 simulations without intersystem crossings, the mean

absolute energy drift was reduced to 0.01 eV.

The energy jumps in the CASSCF calculations often happen when the calculations converge to the wrong active space, representing a discontinuous change of the wavefunctions on the potential energy surface. This issue is controlled by first benchmarking the active space and state-averaging setting to obtain a stable active space covering all major reaction pathways. This active space is used as the initial guess for all training data calculations. On the other hand, it could be difficult to correctly converge the CASSCF calculation for some structures sampled far from the equilibrium regions during the adaptive sampling. Then, we discarded the data from the uncovered calculations. We also noted that a small portion of CASSCF calculations converged to different sets of active orbitals, introducing discontinuous energy data. In our experience, the NN training can smooth out those outliers if the energy jumps are insignificant or the number of outliers is small. Otherwise, the validation errors of NN-predicted energy will be larger than 0.1 eV. Our NNs show validation errors of 0.0337-0.0340 eV in energy, indicating negligible bias from the energy jumps in the CASSCF data.

The CASSCF trajectories show similar energy conservation to the NN trajectories. The trajectories in the S_1 state show an average energy drift of 0.08 eV. The discontinuous PES at the surface hopping point introduced a slight increment of energy drift of 0.006 eV. The accumulations of the energy deviations after multiple surface hoppings resulted in an overall energy drift of 0.29–0.31 eV. Energy conservation can be improved by reducing the simulation time step. For example, the average energy drift of NN trajectories is reduced from 0.15 to 0.07 eV when the time step is lowered to 0.25 fs.

Error analysis

We computed the NN-prediction errors in the S_0, S_1, T_1, and T_2 energies and gradients of the MECIs.

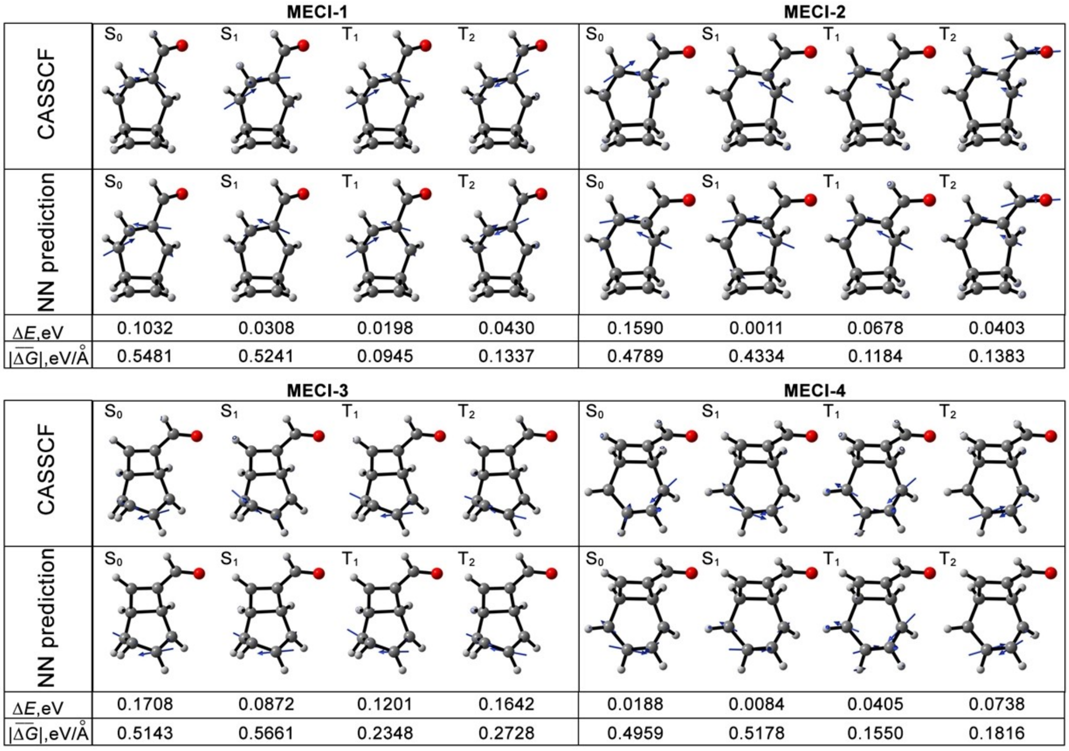

Figure S5. Neural network prediction error analysis at the S_1/S_0 MECI structures. The blue arrows show the gradient vectors.

The S_0 and S_1 of **MECI-1**, **MECI-2**, and **MECI-3** show notable errors larger than 0.10 eV. In all cases, the S_0 and S_1 show larger errors in gradients than T_1 and T_2. The prediction errors of singlet state energies and gradients could lead to inaccurate population transfer from S_1 to S_0, resulting in the underestimated S_0 population in the dynamics. The prediction errors of triplet state energies (0.0198–0.1642 eV) and gradients (0.0945–0.2728 eV/Å) are within 2-3 times of the validation MAEs (0.0373–0.0340 eV and 0.1264–0.1356 eV/Å for energy and gradient), which lead to reliable dynamics after intersystem crossing to T_1 and T_2.

It should also be noted that the most state population transfer to S_0 occurred in the first 200 fs CASSCF dynamics. The following 200 fs CASSCF dynamics show almost no population transferred to S_0 because the competing intersystem crossings to T_1 and T_2 occurred. It suggests the excited-state dynamics of **1** is dominated by the intersystem crossing in a long time scale. To determine the influence of underestimated $S_1 \rightarrow S_0$ transitions on the reaction channels, we turned off surface hopping (i.e., infinitely slow intersystem crossing and still observed the ring-opening reaction of **1** (Table S7). Thus, the underestimated S_0 population in the current ML-photodynamics simulations would not change our conclusions on the ring-opening mechanism of **1**, except for the overestimated ring-opening reaction rate constant via intersystem crossing.

Table S7. Prediction quantum yield of ring-opening reactions in 20 ps ML-photodynamics simualtions at various conditions.

Test cases	Predicted quantum yield of ring-opening
Reference simulations	89%
S_1 dynamics without surface hopping	9% (86% trajectories do not react)
T_1 dynamics without surface hopping	93%
NN-predicted SOC norm – $MAE_{test,SOC}$	86%
NN-predicted SOC norm + $MAE_{test,SOC}$	84%

In addition, we prepared an out-of-sample test set based on the S_1/T_1 surface hopping structures collected from the ML-photodynamics trajectories. Figure 6b shows diverse geometrical distributions of the S_1/T_1 surface hopping structures. Therefore, the test set provides an effective measure of NN prediction accuracy for out-of-sample structures far from the equilibrium geometries (Table S7).

Table S8. Out-of-sample test errors of NN predicted energies, gradients, and SOC norms.

	Validation	Test
MAE_{Energy} (eV)	0.0338	0.1709
$MAE_{Gradient}$ (eV/Å)	0.1310	0.3044
MAE_{SOC} (cm^{-1})	2.1848	13.4160

The test errors are larger than the validation errors as expected. The relatively low validation errors suggest biased error evaluations due to the structural similarity between the validation and training set. These results show the necessity of out-of-sample test for adaptive sampling. One improvement for the future development could be expanding the training and validation set simultaneously by randomly splitting the collected data during

the adaptive sampling. On the other hand, the increased test errors indicate limited extrapolation of feed-forward NNs, which could be circumvented with message-passing graph and equivariant convolutional NNs. The development of equivariant NNs for excited-state potential is also on our to-do list for future works.

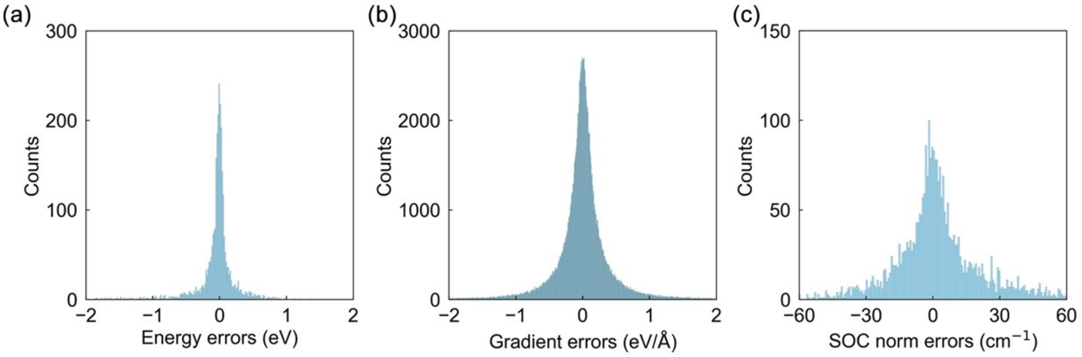

Figure S6. Error distributions of the (a) predicted energies, (b) gradients and (c) SOC norm.

Figure S6 plots the histograms to illustrate the exact differences between the predicted and reference energies, gradients, and SOC norm. The SOC errors show diverse distributions compared to energy and gradient errors, indicating it could either overestimate or underestimate the SOC norm. To determine the sensitivity of ring-opening reaction to the ISC dynamics, we applied a constant bias to the ML-photodynamics simulations by adding and subtracting the MAE (13.4159 cm-1) to the predicted SOC norm to determine the influence of SOC errors on the intersystem crossing (ISC) dynamics. Figure S7a illustrates the state population dynamics using the NN-predicted SOC norm as a reference. The state population dynamics with reduced SOC norms in Figure S7b show results similar to those in Figure S7a. It suggests that the NN overestimations of the SOC norms do not significantly affect the $S_1 \rightarrow T_1$ ISC rates. The state population dynamics with increased SOC norms in Figure S7c illustrate a similar increment of T_1 in the first 5 ps to those in Figure S7a and highlight the $T_1 \rightarrow S_0$ ISC after 10 ps. These results suggest that the NN underestimations of the SOC norms slow down the $T_1 \rightarrow S_0$ ISC. Nevertheless, our ML-photodynamics simulations show that the ring-opening reaction occurs at S_1 and T_1. Thus, the $T_1 \rightarrow S_0$ ISC would be less likely to affect the ring-opening reaction. The predicted yields of **COT** are 86% and 84% for the ML-photodynamics with reduced and increased SOC norms (Table S7), respectively, which are close to the results without modifications of SOC (89%). Thus, the errors in the SOC predictions do not significantly affect our findings in the ring-opening reaction.

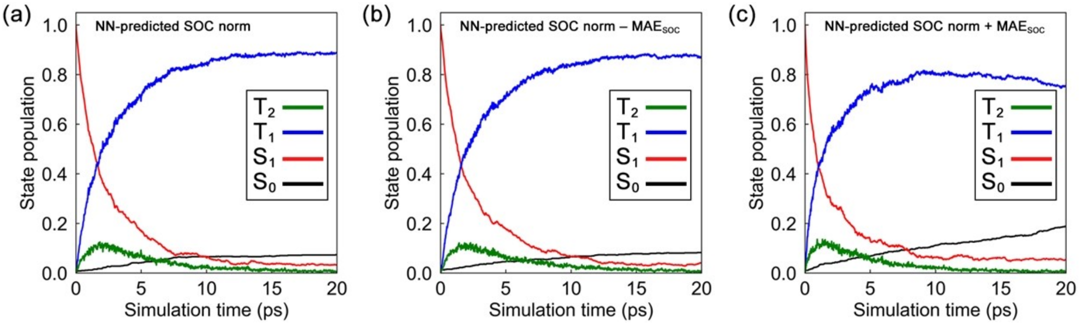

Figure S7. State population dynamics of the ML-photodynamics (a) without modifications of SOC norms, (b) with reduced SOC norms, and (c) with increased SOC norms.

S3. Additional dynamics results

Table S9. Distributions of $S_1 \rightarrow S_0$ and $T_n \rightarrow S_0$ surface hopping points and final products.

	$S_1 \rightarrow S_0$		$T_n \rightarrow S_0$	
	Surface hopping	Final product	Surface hopping	Final product
**1**	2%	0%	4%	0%
**2**	0%	0%	0%	0%
**3**	1%	0%	0%	0%
**4**	0%	2%	0%	4%

Figure S8-S11 collected the trajectories undergoing rare reaction pathways to form the intermediates and product via $S_1 \rightarrow S_0$ and $S_1 \rightarrow T_1$ transitions.

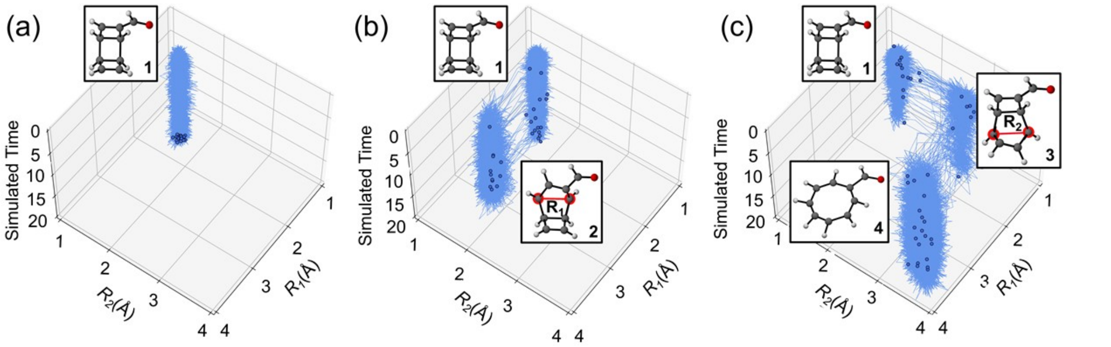

Figure S8. Plots for the ML-photodynamics trajectories undergoing $S_1 \rightarrow T_1$ transitions, where (a) no ring-opening reaction occurred, (b) underwent one ring-opening step to form intermediate **2**, (c) formed **4** in the less favored pathway via intermediate **3**. The black dots represent the latest S_1/T_1 surface hopping points.

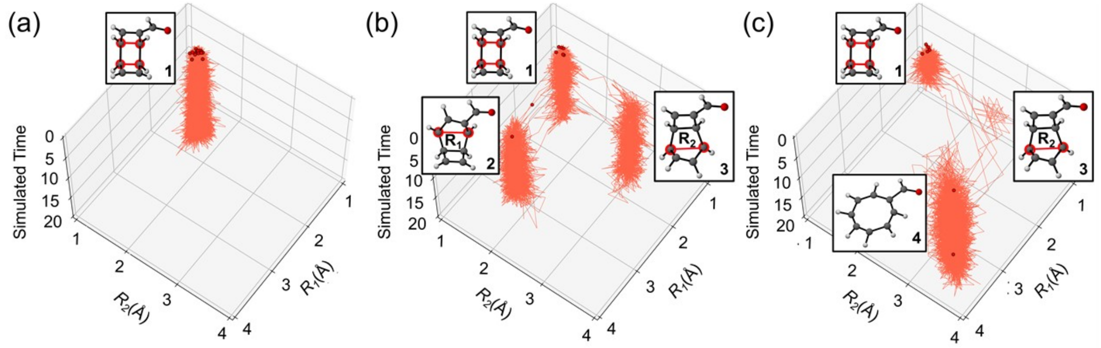

Figure S9 Plots for the ML-photodynamics trajectories undergoing $S_1 \rightarrow S_0$ transitions, where (a) no ring-opening reaction occurred, (b) underwent one ring-opening step to form intermediate **2** or **3**, (c) formed **4** in the less favored pathway via intermediate **3**. The black dots represent the latest S_1/S_0 surface hopping points.

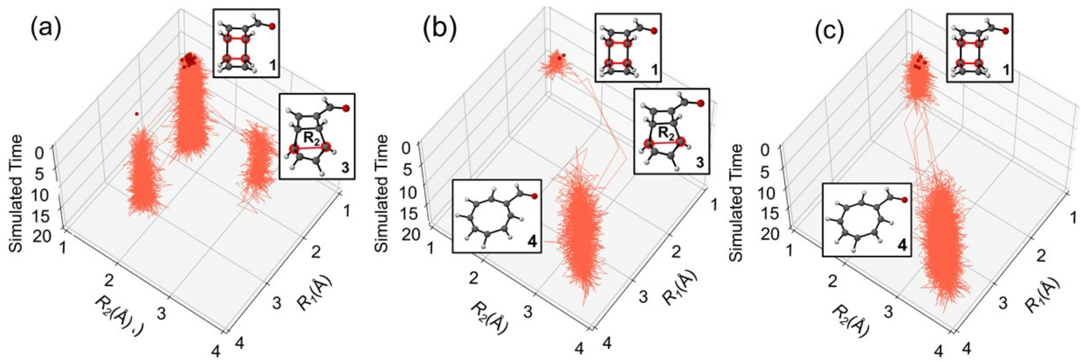

Figure S10. Plots for the ML-photodynamics trajectories undergoing $S_1 \rightarrow S_0$ transitions, where (a) underwent one ring-opening step to form intermediate **2** or **3** in S_0, (b) formed **4** in the less favored pathway via intermediate **3**, (c) formed **4** via a concerted ring-opening process. The black dots represent the latest S_1/S_0 surface hopping points.

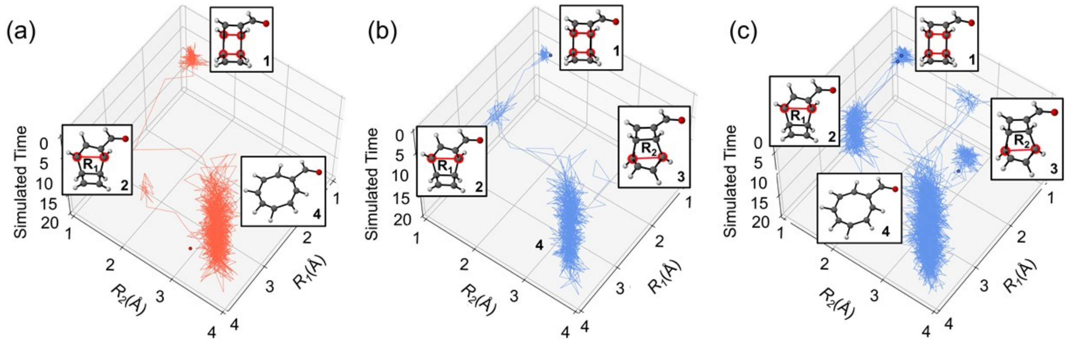

Figure S11. Plots for the ML-photodynamics trajectories undergoing $S_1 \rightarrow S_0$ transitions, where (a) **4** returned to intermediate **2** after the $S_1 \rightarrow S_0$ transition, (b) **4** returned to intermediate **3** after the $S_1 \rightarrow T_1$ transition, (c) **4** returned to intermediate **3** then back to **4** after the $S_1 \rightarrow T_1$ transition.

To determine the influence of intersystem crossings on the reaction channels, we performed 10 ps ML-photodynamics simulations with a time step of 0.5 fs for another 1000 trajectories starting from the S_1-FC point without surface hopping (i.e., infinitely slow intersystem crossing). The simulations show 86%, 5%, and 9% of **1**, **2**, and **4**. The trajectories were trapped in the S_1 minimum region. These results underscore the critical role of intersystem crossing in facilitating the ring-opening reaction of **1**.

We then performed the same simulations above on T_1 to explore the Norrish cleavage. The results show 1%, 6%, and 93% of **1**, **2**, and **4** but no cleavage products. We noted the aldehyde's C-H bond elongation to 1.5 Å in a single trajectory, and the carbonyl group formed intramolecular hydrogen bonding to the γ-H atom of **COT**. These findings indicate possible channels for H detachment and Norrish type II reactions, reported in a previous nonadiabatic dynamics study (J Phys Chem A 2013, 117, 11711−11724). The conjugation between the aldehyde group and **COT** could be a reason for suppressing the Norrish cleavage of **1**. On the other hand, our CASSCF calculations without the active orbitals of the σ_{CC} bond in Norrish cleavage could bias our results, underestimating the ratios for Norrish cleavage. Nevertheless, we do not anticipate the Norrish cleavage would interfere with the ring-opening reaction of **1** because the resulting **TOD** radical will continue the ring-opening reaction to **4**.

S4. Electronic energies and Cartesian coordinates of the optimized geometries

Table S10. The electronic energy of all compounds in the SA5-CASSCF(12,11)/ANO-RCC-PVDZ calculations.

	S_0	S_1	S_2	S_3	S_4
**1**	-420.59236470	-420.46008759	-420.33787917	-420.31870154	-420.30857220
**S_1/T_2**	-420.57466325	-420.46408689	-420.35733839	-420.34360277	-420.34067232
**T_2/T_1**	-420.57407241	-420.45255324	-420.34994867	-420.34807809	-420.33602927
**MECI-1**	-420.48611342	-420.48606312	-420.36768831	-420.36341612	-420.34498085
**MECI-2**	-420.49966669	-420.49964789	-420.42799763	-420.37356700	-420.35705700
**MECI-3**	-420.48986189	-420.48467006	-420.37067481	-420.36422703	-420.34683418
**MECI-4**	-420.49330429	-420.49328601	-420.38065924	-420.36986795	-420.35328114
**2**	-420.67700754	-420.53347677	-420.45521304	-420.43172927	-420.42044360
**3**	-420.68127757	-420.53626375	-420.43751066	-420.43381555	-420.43318738
**4**	-420.70324806	-420.54954722	-420.48310250	-420.47281349	-420.45723325
	T_1	T_2	T_3	T_4	T_5
**1**	-420.46727043	-420.46193493	-420.43563723	-420.38637734	-420.33972697
**S_1/T_2**	-420.46966509	-420.46403927	-420.46029309	-420.38602482	-420.35566915
**T_2/T_1**	-420.46786307	-420.46785070	-420.45428427	-420.37398806	-420.35481223
**MECI-1**	-420.50431752	-420.43314087	-420.41595486	-420.37350399	-420.36832728
**MECI-2**	-420.51441946	-420.43011874	-420.41572858	-420.41123123	-420.36476343
**MECI-3**	-420.50666084	-420.40486492	-420.39750625	-420.37062153	-420.36078742
**MECI-4**	-420.51190513	-420.44314291	-420.41915378	-420.37544805	-420.37141733
**2**	-420.57420127	-420.54115739	-420.51472057	-420.51224304	-420.46381734
**3**	-420.56711522	-420.54577271	-420.54417436	-420.49699676	-420.46635700
**4**	-420.58803942	-420.55980716	-420.55914249	-420.54986976	-420.52606011

Cartesian coordinates of optimized structures

1

C	-2.954658250	7.819567780	-0.049759450
C	-1.632256230	8.360632410	0.595320030
C	-3.781533750	8.341149480	1.212942300
C	-0.911130810	7.481590460	1.593170790
C	-2.460301580	8.882560100	1.854678750
C	-1.609386170	7.924150980	2.656191350
H	-2.475887300	9.926460460	2.136461690
H	-3.166075620	8.161206850	-1.053804190
H	-1.050836430	9.029602740	-0.024853350
H	-4.592076180	9.049482470	1.119461880
H	-0.162109550	6.720046010	1.473633880
H	-1.624472340	7.645068940	3.693639630
C	-3.428671610	6.443701430	0.358293280
H	-3.217642390	5.460047110	-0.020694280
C	-4.127314340	6.885070360	1.426000880
C	-4.868012350	6.190743760	2.479621770
O	-5.358276990	6.757787960	3.425843400
H	-4.960917660	5.108489070	2.381732030

S_1/T_2 MECP

C	-2.933462255	7.855060358	-0.046838108
C	-1.614850533	8.405838331	0.590606293

C	-3.761297655	8.357184013	1.222231865
C	-0.853548329	7.501202235	1.530516391
C	-2.447161783	8.904332708	1.867349008
C	-1.613369482	7.953508451	2.692846106
H	-2.484582843	9.942815325	2.170806088
H	-3.154837175	8.197897052	-1.049114195
H	-1.048282170	9.081753198	-0.037722395
H	-4.581038766	9.056956423	1.137607607
H	-0.256181728	6.627222394	1.353384245
H	-1.774865134	7.524242676	3.662921022
C	-3.384779359	6.467494729	0.348821313
H	-3.157476823	5.491070299	-0.037407349
C	-4.092367103	6.894017926	1.437821862
C	-4.826013277	6.211262019	2.447223443
O	-5.394960127	6.813855579	3.400143798
H	-4.920605255	5.134830372	2.414756228

T_2/T_1 MECI

C	-2.932012029	7.857308054	-0.050247819
C	-1.616086591	8.409089162	0.587998952
C	-3.762428317	8.359184186	1.222951261
C	-0.856703845	7.498401396	1.525235435
C	-2.448834269	8.907030798	1.864930250
C	-1.619857619	7.952411265	2.692540633
H	-2.486763419	9.944520562	2.171220190
H	-3.149716676	8.194607400	-1.055428513
H	-1.046771014	9.083536065	-0.039412571
H	-4.586553967	9.054799592	1.148737887
H	-0.277969406	6.612781332	1.343027886
H	-1.797359268	7.511310198	3.654521133
C	-3.364249710	6.461997720	0.335173011
H	-3.121256296	5.488225104	-0.044672365
C	-4.093061717	6.902371481	1.461852754
C	-4.838756426	6.227528859	2.478222699
O	-5.389686141	6.811905899	3.401891739
H	-4.911613100	5.143534999	2.407410650

MECI-1

C	-2.256410470	7.268140780	0.677824120
C	-1.885567920	8.733555230	0.656634540
C	-3.698036030	8.293823110	2.337185390
C	-0.683502150	9.134126430	1.497198130
C	-2.754199490	9.356921350	1.831976690
C	-1.417999960	9.605915100	2.514522740
H	-3.294400660	10.257713920	1.567988010
H	-2.678709370	6.801988300	-0.195512180
H	-1.989307620	9.178661920	-0.326824370
H	-4.749913420	8.447509560	2.176669730
H	0.372333720	9.016578980	1.341103930
H	-1.172086660	10.010736690	3.478368650

C	-2.008813600	6.466282590	1.874142470
H	-1.709219530	5.435031370	1.854978270
C	-3.254825710	6.848342210	2.348318870
C	-4.227422320	5.848077470	2.841435670
O	-5.356016100	6.115468590	3.173575460
H	-3.867504490	4.820241870	2.891606810

MECI-2

C	-2.417332270	7.819097410	-0.112074320
C	-1.905019090	9.040959010	0.618830700
C	-3.767940210	8.077882580	1.776165670
C	-0.800952110	8.902021360	1.656652790
C	-2.861271280	9.283129610	1.856014080
C	-1.606991460	9.136466280	2.701712780
H	-3.406281680	10.219214320	1.900152150
H	-2.570099240	7.865603010	-1.174571600
H	-1.807694250	9.885555900	-0.050912130
H	-4.823692460	8.218674100	1.609783940
H	0.242216240	8.664336630	1.566131540
H	-1.454942390	9.168095340	3.763298630
C	-2.957399790	6.660236910	0.603659220
H	-3.285899900	5.827200950	-0.005000550
C	-3.260761540	6.738376550	1.959528830
C	-3.826577060	5.689407080	2.762104940
O	-4.533873770	5.854697180	3.724700830
H	-3.557252780	4.675519810	2.451064930

MECI-3

C	-2.861038770	8.009945590	0.046731670
C	-1.626843280	8.840904080	0.320237900
C	-3.770306980	8.272596890	1.319818660
C	-0.789108500	8.558180850	1.488655240
C	-3.096476840	9.333549010	2.150599330
C	-1.590681910	9.404532920	2.220016700
H	-3.626250710	10.254770050	2.318683300
H	-3.299821300	8.204040000	-0.925736170
H	-1.327197270	9.599865350	-0.381339740
H	-4.800570410	8.527394720	1.109494280
H	0.275636850	8.696607560	1.482159280
H	-1.196622680	10.200384390	2.839439080
C	-2.813057440	6.564835980	0.518672170
H	-2.324011690	5.691221840	0.130395510
C	-3.543359960	6.798704090	1.626239560
C	-3.962866800	5.944917950	2.737316110
O	-4.645128020	6.336266610	3.650008580
H	-3.627471590	4.907332310	2.707030440

MECI-4

C	-2.789911070	8.022698430	-0.018399660
C	-1.518214940	8.802903240	0.048897510

C	-3.710438380	8.333742040	1.276382240
C	-1.246577930	9.621693900	1.261634410
C	-3.114742930	9.375079920	2.152767780
C	-1.695225430	9.314847380	2.563127840
H	-3.629732830	10.314753400	2.251357100
H	-3.311417430	8.125434830	-0.964971340
H	-0.641983920	8.360392710	-0.402255770
H	-4.738331830	8.567923520	1.028764000
H	-0.622405200	10.489732840	1.104951080
H	-1.376541050	9.971311420	3.355998860
C	-2.722986030	6.602942950	0.523639190
H	-2.236562520	5.716936790	0.159639510
C	-3.470505710	6.868808610	1.610282490
C	-3.932203320	6.038628410	2.723652150
O	-4.673715690	6.440788110	3.582942090
H	-3.571044580	5.009415850	2.744674090

2

C	-2.28218932	7.75661455	-0.32438109
C	-1.99299712	8.92618329	0.57047442
C	-3.98099783	7.98013751	2.01581857
C	-0.87127435	8.75506431	1.57747493
C	-2.94261519	9.05023371	1.86696006
C	-1.66103134	8.85752931	2.65618584
H	-3.39272090	10.03228196	1.98039151
H	-1.68470233	7.64469945	-1.21333630
H	-1.93518170	9.84515728	-0.00603645
H	-4.64438878	8.02856196	2.86122573
H	0.18462220	8.60318809	1.44818785
H	-1.47713075	8.81749382	3.71401049
C	-3.24225180	6.85795767	-0.04600269
H	-3.40578631	6.02984883	-0.71413070
C	-4.10515369	6.96296652	1.13787434
C	-5.14158706	5.91903508	1.33546851
O	-5.90768880	5.87499342	2.26168264
H	-5.19002352	5.14512917	0.56683420

3

C	-3.27058392	8.23492940	0.01705889
C	-1.98969813	8.78221332	0.57676190
C	-4.55082996	8.31709443	0.99701110
C	-1.91054151	9.27995312	1.82366541
C	-4.29213157	8.91873127	2.34785434
C	-3.07203268	9.34691853	2.71799601
H	-5.12405516	8.99066478	3.02624870
H	-3.46478361	8.64246934	-0.97084124
H	-1.11869880	8.76508415	-0.05651770
H	-5.42058008	8.76970034	0.53177794
H	-0.97208854	9.65887480	2.18855284
H	-2.92803824	9.76723494	3.69765667

C	-3.46482740	6.73310305	0.10038981
H	-2.92407570	5.91357447	-0.33727224
C	-4.53688476	6.80172475	0.91405812
C	-5.39406111	5.77650665	1.50608042
O	-6.34576379	6.02990100	2.20186883
H	-5.13585196	4.74008635	1.28488392

4

C	-2.291770160	8.104099690	-0.760851860
C	-1.146887900	8.024807280	0.168005500
C	-3.994497520	8.495560780	1.978577780
C	-0.953955100	8.761361090	1.271236270
C	-3.147509400	9.691509080	2.112621220
C	-1.844078050	9.809057170	1.808997900
H	-3.630553660	10.536984150	2.575753840
H	-2.032614310	8.223145660	-1.800921470
H	-0.368163980	7.337182880	-0.121099990
H	-4.565827340	8.230489770	2.852087310
H	-0.030092510	8.624344130	1.810391180
H	-1.362543560	10.745605470	2.040716810
C	-3.593445880	7.981574150	-0.458509300
H	-4.296813860	8.007563300	-1.276332300
C	-4.188627700	7.741929000	0.875385620
C	-5.155090530	6.610316050	0.935388300
O	-5.734468320	6.249990380	1.925814590
H	-5.327874850	6.087109630	-0.006884620

S5. References

1. Abadi, M.; Agarwal, A.; Barham, P.; Brevdo, E.; Chen, Z.; Citro, C.; Corrado, G. S.; Davis, A.; Dean, J.; Devin, M., Tensorflow: Large-scale machine learning on heterogeneous distributed systems. *arXiv preprint arXiv:1603.04467* **2016**.
2. Shu, Y.; Zhang, L.; Chen, X.; Sun, S.; Huang, Y.; Truhlar, D. G., Nonadiabatic dynamics algorithms with only potential energies and gradients: Curvature-driven coherent switching with decay of mixing and curvature-driven trajectory surface hopping. *Journal of Chemical Theory and Computation* **2022,** *18* (3), 1320-1328.
3. Zhao, X.; Merritt, I. C.; Lei, R.; Shu, Y.; Jacquemin, D.; Zhang, L.; Xu, X.; Vacher, M.; Truhlar, D. G., Nonadiabatic coupling in trajectory surface hopping: accurate time derivative couplings by the curvature-driven approximation. *Journal of Chemical Theory and Computation* **2023,** *19* (19), 6577-6588.
